# A field study to evaluate PMI estimation methods for advanced decomposition stages

**DOI:** 10.1007/s00414-020-02278-0

**Published:** 2020-04-05

**Authors:** Stefan Pittner, Valentina Bugelli, Katharina Weitgasser, Angela Zissler, Sangob Sanit, Lena Lutz, Fabio Monticelli, Carlo P. Campobasso, Peter Steinbacher, Jens Amendt

**Affiliations:** 1 Department of Forensic Medicine, University of Salzburg, Salzburg, Austria; 2 Department of Medicine and Health Sciences, University of Molise, Campobasso, Italy; 3 Department of Biosciences, University of Salzburg, Salzburg, Austria; 4 Department of Parasitology, Chiang Mai University, Chiang Mai, Thailand; 5 Institute of Legal Medicine, Goethe-University Frankfurt, Frankfurt, Germany; 6 Department of Experimental Medicine, University L. Vanvitelli of Campania, Aversa, Italy

**Keywords:** PMI estimation, Entomology, Protein degradation, Morphology, Total body score, Field study

## Abstract

Estimating the postmortem interval (PMI) is one of the major tasks and a continuous challenge in forensic pathology. It is often an exclusion process of available methods, which ultimately can lead to an unsatisfactory outcome due to poor reliability. This problem is most acute in the late PMI, when decomposition proceeds and some methods (such as rigor, livor, and algor mortis) are no longer applicable. Several methods, such as forensic entomology, skeletal muscle protein degradation, and the study of body decomposition by application of a morphological scoring, are expected to provide further information; however, all have certain limitations and weaknesses. Availability of a tool-box of methods allows a case-specific selection of the most appropriate one(s), or eventually provides improvements in the overall accuracy and precision of the PMI estimation by merging and combining methods. To investigate practical (field) application, eventual interferences, and/or synergetic effects, as well as the robustness of these methods towards specific influencing factors, a field study was conducted, using eight pig cadavers of different body weights and physical coverage, left to decompose under natural conditions for 16 days. Morphological changes during decomposition were assessed using the total body score (TBS), muscle samples were collected to analyze protein degradation, and insect colonization was evaluated. The results reveal strengths and current limitations of all tested methods, as well as promising synergistic effects, and thus, provide a baseline for targeted future research.

## Introduction

To estimate the time since death, or postmortem interval (PMI), as precisely as possible is a central, yet very complex aspect in forensic pathology [1]. Most of the available methods to date, such as the comparison of body core and environmental temperature [2], analysis of *rigor* and *livor mortis* [3], or the assessment of supravital phenomena [4], are more or less restricted to specific circumstances of death, dependent on the stage of decay or limited to certain postmortem timeframes. However, in early-stages postmortem, a combination of these approaches allows a more reliable estimation of the PMI [5, 6]. In later stages, the life cycle of necrophagous insects developing on the body [7], the analysis of tissue degradation [8–10], or morphological changes [11] of a dead body can contribute to exclude specific timeframes or provide minimum postmortem intervals (PMI_min_). However, these methods can only be applied under specific circumstances (mere presence of insects, or remaining tissue, no exclusion criteria such as myiasis [12, 13], or altered in vivo tissue metabolism [14], etc.) and only little is known about how the investigated phenomena influence each other. In fact, PMI estimation in forensic practice is often an exclusion process of available methods, which ultimately can lead to unsatisfactory outcome due to poor reliability and/or accuracy. This problem is most acute in the late PMI, when decomposition advances and most of the abovementioned methods are no longer useful.

Human decomposition is a very complex process due to the interaction of different biological and chemical components [15]. Up to date, a unique decomposition model cannot match all potential forensic cases because of highly variable decomposition processes taking place in response to the effect of extrinsic- or intrinsic-influencing factors. Extrinsic factors include environmental conditions (e.g., temperature, moisture, clothing, concealment, weather, and insect activity), while intrinsic factors are mostly related to the cause and manner of death and the characteristics of the human body (body mass, age, gender, injuries, or medical treatments) [15–19]. Of these, temperature and humidity are considered among the most significant variables affecting the rate of decomposition [20].

Currently, several methods are expected to assist in the estimation of the PMI in the advanced stages of decomposition such as forensic entomology [21], the skeletal muscle protein degradation [10], and the classification of decomposition morphology by the means of a total body score (TBS) [11]. However, the reliability of these methods is also affected by several sources of inaccuracy, mostly related to biotic (bacteria, plants, scavengers) and abiotic (rainfall, ventilation, soil) factors. Moreover, they are validated to varying degrees. Temperature, environment, and access for insects are well known as the most important variables influencing degradation or preservation of soft tissues [21, 22]. Hence, forensic entomology, e.g., although a well-established method of estimating the PMI via the age of the insects developing on the body, is only capable of providing a *minimum* PMI (PMI_min_) as insects do not always have immediate access to the corpse and might colonize it with delay. Succession of insect species occurring on cadavers can follow a regular pattern and as such be used as a manual for timeline data which, depending on the case, can additionally narrow down time period [23]. However, application can be delicate, as some species depict a random occurrence pattern and do not necessarily follow the predicted succession [24]. Moreover, community structure and arrival patterns as well as associated decomposition rates can as well alter the access for insects [25]. The study of skeletal muscle protein decay is promising [10] and offers some advantages: its immediate use following death, the high abundance of muscle tissue in human bodies, and the ease of sampling. Although this method has already proven its potential in human application [26, 27], it requires additional validation, especially on behalf of possible influencing factors. Last but not the least, decomposition scoring systems are still of little help in forensic practice [28, 29]. In fact, the regression equations developed from such decomposition scores seem to be useful only if applied to bodies exposed to exactly the same experimental conditions as to which they have been tested.

In case work, there is often a certain time point of interest, such as if a person died before or after a specific date. In combination with the numerous variations of the circumstances of death, this requires a tool-box of methods to select the most appropriate ones for case-specific application. Some methods have to be excluded under certain circumstances, under which other approaches work just fine. Ultimately, merging and combining several methods can improve the overall accuracy of the estimation, just as used in the compound method for early-period PMI estimation [5, 6].

Aiming to develop a similar concept for advanced decomposition, a pilot study was designed using eight pigs of varying body weights and different forms of physical coverage. The pigs were placed in a rural area and left to decompose under natural conditions: (i) morphological changes were classified and assessed using TBS, (ii) muscle samples were collected and their protein degradation analyzed, (iii) insect colonization was evaluated and their immature development recorded. Simultaneously, environmental conditions were monitored, so that changes could be evaluated in context to accumulated degree days (ADD) over the course of 16 days of decomposition.

## Material and methods

### Animals and experimental design

**Fig. 1:**
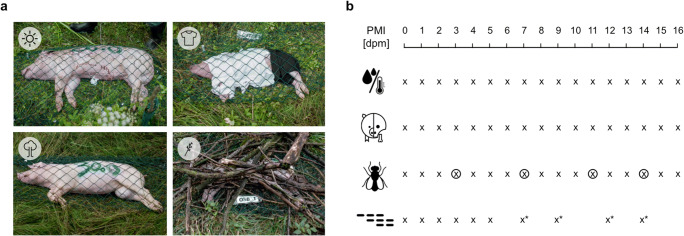
Experimental setup. **a** Eight pigs were randomly allocated to four treatment groups. Top left: open glade naked; top right: open glade clothed; bottom left: in the shadow of large trees; bottom right: covered with branches and twigs. **b** Over the course of 16 days postmortem (dpm), environmental conditions were monitored, morphology was assessed by a total body score (TBS), entomology samples were collected and analyzed, and skeletal muscle samples were taken and investigated for protein decomposition. Icons indicate (from top to bottom) measurement of environmental conditions; morphology assessment; entomology assessment; and sampling of muscle tissue. “x” indicates a sampling/observation day, circled “x” indicates the sampling of insect larvae on the “days of discovery,” “x*” indicates sampling with reduced sample size (*n* = 4) due to loss of tissue

Eight pigs with a body weight between 18 and 66 kg were killed within 30 min and transported to a forensic research field in northern Germany (N 51.915, E 7.907) in summer (July–August). The cadavers were randomly allocated to one of four treatment groups, covered with wire mesh to prevent vertebrate scavenging and equipped with two temperature sensors, one internal (rectal) and one within 1-m distance to the pig. Four pigs were placed in pairs at an open glade with moderately high grass and small bushes nearby: two animals (66 and 57 kg) were placed on the ground surface naked, while the other two (19 and 18 kg) were placed at 30-m distance to the first pair and dressed with shirts and pants. The other four pigs were placed in pairs in a forest area: two (30 and 26 kg) were placed without any direct coverage, but in the shade of large, sparse trees, approximately 400 m away from the forest glade of the first two pairs. Two carcasses (25 and 18 kg) were placed again at an open meadow-like area, about 50 m away from the pigs underneath the trees, and were covered with branches and twigs. The two pigs of one experimental group were always placed about 10 m apart each other (Fig. 1a).

The carcasses were left to decompose under natural conditions for a total of 16 days. A weather terminal (IButton® Thermochron, maxim integrated) was used to monitor temperature and humidity in 60 min intervals. Each day, a morphological score (TBS) was assessed and insect activity was checked. At 4 days (3, 7, 11, and 14), insects were collected, and species and developmental stages were identified. Tissue samples were collected on days 0, 1, 2, 3, 4, 5, 7, 9, 12, and 14. The first assessment and sampling took place approximately 2 h after the animals were killed. These samples are further referred to as day 0. All additional samplings took place every 24 ± 1 h after the first sampling and are from here on referred to as day 1 to day 16 (Fig. 1b).

### Morphology

Each day, a total body score (TBS) according to Megyesi et al. [11] was evaluated by at least two independent assessors to reduce possible observer bias. The three body regions (i) head and neck, (ii) trunk, and (iii) limbs were individually scored with 1 (“fresh, no discoloration”) to a maximum of (i) 13, (ii) 12, and (i) 10 points (dry bone). These values were added to obtain the TBS for all the carcasses on each day of the sampling period. Additional details and morphology alterations that are not included in the scoring scheme were documented (e.g., “no bone exposure, but broken skin at the abdomen,” “difficult to assess due to large larval mass”). Digital photos were taken for documentation purposes and to discuss assessments with other authors, again to reduce observer bias. SPSS 23.0 software (IBM Corp.) was used for statistical analysis.

### Entomology

Every day, each carcass was evaluated for 15 min. During this period, flying insects were sampled with the help of an insect net and the general insect colonization of each carcass was described. To reduce a possible destructive impact on the experimental design (e.g., by removing the brushwood for extensive sampling) and the associated examinations (e.g., estimating the TBS), larval samples were mainly collected from the carrion surface and/or the region between body and ground. For the same reasons, and to avoid to disturb the colonizing fauna, 4 days were chosen as “days of discovery” (days 3, 7, 11, 14), where the sampling of larvae at a crime scene was simulated. Inexperienced assessors received a short practical training in sample collection and treatment, which reflects the situation in practical routine very well. A special focus was put to the pig’s heads and anogenital regions to check for insects. If present, eggs and larvae were collected with tweezers and small spoons and partly reared on minced meat in an outdoor breeding on site to reach the adult stage and/or killed in hot water and then stored in 70% ethanol for later measurement and identification. Since the first fly larvae migrated away from the carcasses after about 1 week, soil samples were taken from the immediate vicinity of the carcasses on days 11 and 14 and checked for the presence of larvae and pupae. Where available, the specimens were further bred in the same outdoor breeding mentioned above. The identification of the larval and adult stages was carried out with the keys of Grzywacz et al. [30], Rognes [31], Szpila et al. [32, 33], Pape [34], Gregor et al. 2002, and Freude [35] and the use of a ZEISS stereomicroscope. Measurements of larval size for age estimation were performed using a geometric micrometer [36].

### Protein analysis

Tissue samples from *M. biceps femoris* were extracted by muscle biopsy. A small incision (approximately 5 mm) through the skin and muscle fascia was made using a scalpel blade. A 5-mm diameter biopsy needle was inserted to a depth of approximately 4–5 cm and muscle tissue was collected until an overall sample size of 5 × 5 × 5 mm was obtained. The tissue material was transferred into a vial tube with 1 mL of extraction buffer (RIPA buffer (SIGMA), together with a protease inhibitor cocktail (ROCHE)) and stored in a cooling box until further processing. After sampling, the wounds were sealed with cyanoacrylate glue (Loctite super glue) to avoid additional potential entry sites for insects and bacteria. For each subsequent sampling, a minimum distance of 2 cm from the previous sampling sites was maintained in order to avoid interferences.

A two-step process was applied to homogenize the samples. After the tissue material was dispersed using an Ultra Turrax (IKA Werke GmbH & CO. KG), all samples were additionally broken down by high-frequency sonication (Hielscher Ultrasonics GmbH) and centrifuged at 1000×*g* for 10 min. The supernatant was collected and stored at − 20 °C for further analysis. Overall protein concentration was determined by BCA assay in order to dilute all samples to the same concentration.

Electrophoreses (SDS-PAGE) were run on 10% polyacrylamide resolving gels and 5% polyacrylamide stacking gels, according to a standard protocol [10]. A total of 30–60 μg of protein (depending on the analyzed protein) was prepared, denatured at 90 °C for 5 min, and inserted into the gel wells. Proteins in the gels were transferred onto polyvinylidene fluoride (PVDF) membranes and stored at − 20 °C. Membranes were blocked in blocking buffer, then incubated with primary and secondary antibodies. Between each antiserum incubation step, the membranes were rinsed and extensively washed in washing buffer (3 × 10 min). Primary antisera against the following proteins were used: cardiac troponin T, desmin, tropomyosin, and vinculin. HRP-conjugated polyclonal goat anti-mouse immunoglobulins were used as secondary antiserum. Staining was visualized by addition of chemiluminescence substrate and documented using a digital gel analysis system (Fusion FX7, Peqlab Biotechnology). Protein band intensities were measured using ImageJ software (ImageJ 1.45s, Java 1.6.0_20). Bands from the samples collected at day 0 were considered the native form. All alterations (disappearance of the native bands or appearance of additional bands) were considered degradation events. Signals < 1% of the intensity of the native bands were considered background and thus no band.

The correlation of changing presence/absence of protein bands with increasing PMI was tested using Spearman’s *ρ* (*p* values < 0.05 were considered statistically significant). Logistic regressions were calculated to investigate the temporal dependence (changing probability of band presence over time) for all significant alterations. SPSS 23.0 software (IBM Corp.) was used for statistical analysis.

## Results

### Environmental conditions

Ambient temperature and weather conditions are the most important influencing factors on most decomposition processes and insect colonization. Therefore, the indirect effects of the existing (mostly unpredictable) weather conditions (sun/shade, coverage/clothing, microclimate) were monitored in course of the present experimental setup.

**Fig. 2:**
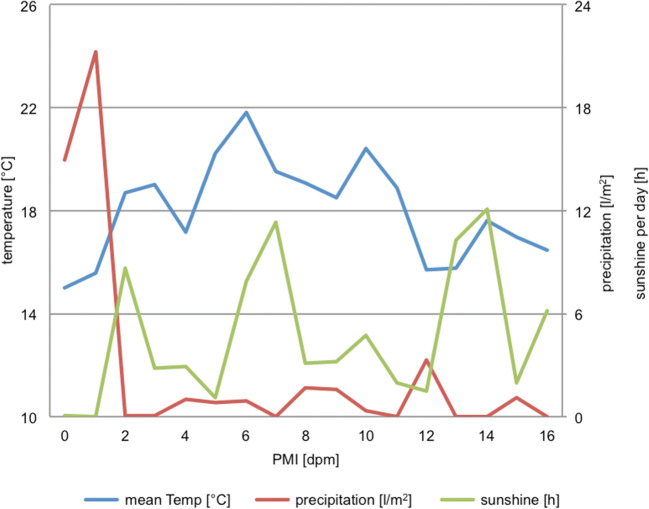
Weather conditions during the experimental time period. Daily mean temperatures, total precipitation, and hours of sunshine per day are plotted

During the first 2 days, there was a lot of precipitation (15.0 and 21.3 l/m^2^ respectively) and (compared with the mean throughout the experiment) low temperatures of 15.0 and 15.6 °C, followed by a phase of dry and moderately warm days until day 11 (mean 0.7 l/m^2^ and 19.5 °C). After that, the weather conditions were largely unstable with some rainy days and a mean temperature of 16.0 °C until the end of the experiment. Cloud coverage and hours of sunshine per day alternated almost periodically each week between 0 and 2 and 10 and 12 h per day (Fig. 2). This represents the conditions of a mild temperate summer climate, typical for the selected area. Temperature conditions, with a mean of 18.02 °C, almost exactly met the climate average of 18.0 °C; however, 95.5 L/m^2^ (extrapolated to monthly precipitation) rainfall was higher than average for this region (77.0 L/m^2^). Reference data were downloaded from the German weather service (www.dwd.com, Oct. 2019).

### Morphology

To make sure to assess morphology changes precisely, it is important to observe the whole body surface. However, due to the experimental setup (the pigs were not supposed to be moved), some difficulties occurred. Coverage, clothing, and branches, but also large larval masses, partly affected the determination of a morphology score. In these cases, a compromise had to be found individually, to avoid disrupting ongoing processes and still uncover as much as necessary to allow a reliable evaluation.

**Fig. 3:**
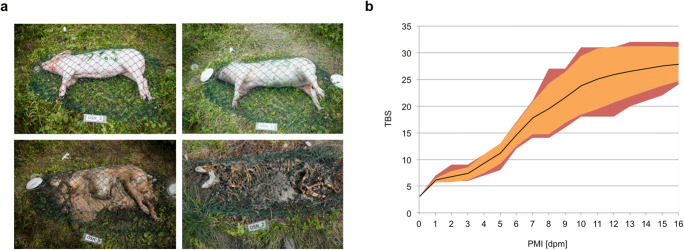
**a** Progression of morphological changes during the decomposition of a pig. Top left: “fresh” day 0, TBS 3; top right: “bloating” day 4, TBS 9; bottom left: “advanced decomposition” day 10, TBS 27; bottom right: “skeletonization” day 16, TBS 32. **b** Development of TBS scores during the course of 16 days postmortem (dpm). Central solid line indicates the mean TBS; orange area represents the mean ± SD, red area the minimum and maximum TBS at each investigated time point

Therefore, a TBS assessment of each pig on every day throughout the experimental period was obtained. All carcasses underwent morphological changes according to the expected succession and within 16 days, all reached more or less skeletal stages, varying predominantly in the remains of grease, mummified, or decomposed tissue (Fig. 3a).

The mean TBS of all carcasses constantly increased from day 1 to day 16. A linear regression of the mean TBS over PMI resulted in the following equation: TBS = 1.71 PMI + 2.33, with an *R*^2^ of 0.96. However, the range of individual TBS values at a specific day increased up to 13 points on day 12 (minimum TBS 18, maximum TBS 32) before it decreased again to 8 points on day 16 (min. 24, max 32). Moreover, while a TBS of 3 was only observed on day 1, a TBS of 7 was assessed (on different animals) between day 2 and day 4, a TBS of 17 between day 6 and 9, and a TBS of 27 between day 8 and 16 (Fig. 3b). No differences or trend was detected on behalf of the influencing factors body weight and exposure towards more advanced or delayed morphological changes.

### Entomology

**Fig. 4:**
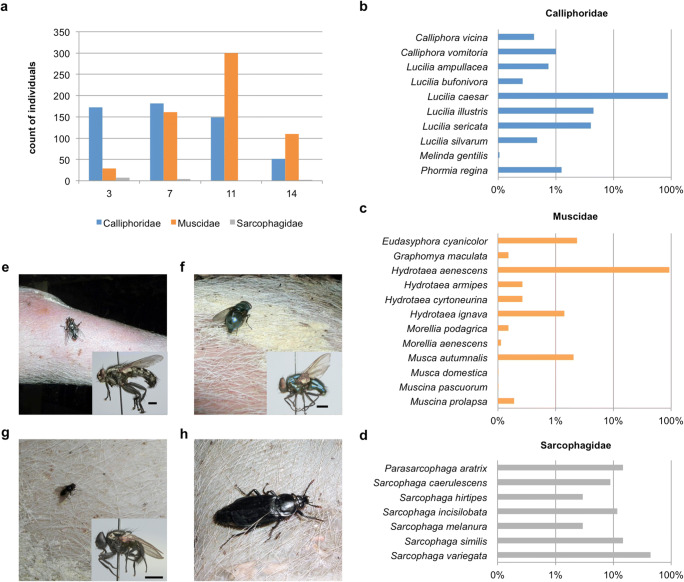
**a** Number of individuals per family caught on the “days of discovery.” **b**–**d** Species diversity within the three major families. Bars indicate the percentage of the total catch per family on a logarithmic scale. In total, 4557 animals of 29 different species were counted. **e**–**h** Adult stages of the four most abundant species found on the pig cadavers: *Sarcophaga variegata* (**e**), *Lucilia caesar* (**f**), *Hydrotaea aenescens* (**g**), *Necrodes littoralis* (**h**)

During the investigated time period, a total of 4557 adult flies were collected, which belonged to 29 species, distributed among three families (Fig. 4). However, the vast majority of animals belonged to two fly species: *Hydrotaea aenescens* (Muscidae) represented 53.6% and *Lucilia caesar* (Calliphoridae) 36.3% of all adult catches, respectively.

Despite the heavy rain during the first days after placement of the carcasses, flies colonized seven out of eight until day 2, but just two of them (one of the naked and one of the clothed carcasses in the open glade) were colonized on day 1. One of the two pigs placed under brushwood showed no signs of immature insect activity at all during the first days and was initially colonized only on day 4. A substantial infestation by fly larvae developed in the following days, peaking on days 7 to 9, especially for the easier accessible carcasses, while the amount of infestation of the carcasses placed under brushwood was much lower. The first migrating, post-feeding maggots occurred on day 8 for the naked pigs in the open glade. From day 12 at the latest, all carcasses showed migrating larvae in the surroundings, but already on day 11 the first pupae were found in soil samples around the carcasses in the open glade and beneath the brushwood. Being present already from day 3 on some of the carcasses, carrion beetles (Coleoptera: Silphidae) and their larvae dominated from day 15 onwards. Here, especially a large number of *Necrodes littoralis* specimens were present.

As with the adult stages, blow flies largely dominated the samplings—none of the four sampling days revealed any colonization by Muscidae or Sarcophagidae on the pig carcasses until day 16 postmortem. Moreover, the blow fly infestation was distinctly dominated by one species, *L. caesar*. With the exception of the species *L. bufonivora* and *Melinda gentilis*, every blow fly taxon was detected in its larval stage on several carcasses, but was outcompeted by *L. caesar*. Few blow fly taxa (such as *Calliphora vicina*, *C. vomitoria*, and *Phormia regina*) in their juvenile stages have been found on one of the four sampling days on the carcasses*.*

The four sampling days, defined as “days of discovery,” showed the following results:

- On day 3, there were only second instar larvae on the carcasses. However, as stated above, this did not apply to all pigs in the same way. While some carcasses just showed first instar larvae, one was not colonized at all.
- On day 7, all carcasses showed infestation by blow fly larvae and, with exception of the one carcass that was not colonized before day 4, the developed larvae were predominately in their third instar.
- On day 11, the majority of carcasses showed highly advanced insect infestation with fly larvae at different stages of development and also several larvae of Coleoptera. All carcasses showed post-feeding fly larvae and the soil samples from three pigs revealed fly pupae.
- On day 14, most carcasses were dominated by adult and larval beetles, while fly larvae activity had declined due to post-feeding migration.

Analyzing the insect fauna = fly larvae on day 3 leads to a PMI_min_ of about 2 days (based on the developmental time of egg and first larval instar) regardless of fly species taken as a basis. Until this early stage of the life cycle, the development of many forensically important fly species is quite similar, at least within a window of about 12–24 h. For the remaining days (7, 11, and 14), all samples were identified on species level and were clearly dominated by *L. caesar*, a blow fly species about which there are almost no developmental data published to date. Based on a mean ambient temperature of about 18 °C (until the first adult flies were hatching in the outbreeding) and using the unpublished data of Richards, Rowlinson, and Hall (Hall, personal communication), it was possible to estimate a period of development for the days of discovery 7 and 11, leading to a PMI_min_ of about 5 and 10 days for all carcasses, with exception of the one that was not colonized before day 4. This would imply a latest date of colonization at day 2 after the placement. On day of discovery 14, the soil samples still contain many pupae of *L. caesar*. Rearing those specimens until the adult stage and calculating as described above resulted in a PMI_min_ of about 12 days. That again indicated a latest date of colonization at day 2 after the placement of the pig carcasses.

Adults of the carrion beetle *N. littoralis* (Coleoptera: Silphidae) were present already after 2 days. However, assuming an average temperature of approximately 18 °C and referring to Matuszewski et al. [37], this would suggest a PMI_min_ of 6 days, and thus an overestimation of about 4 days.

### Protein analysis

**Fig. 5:**
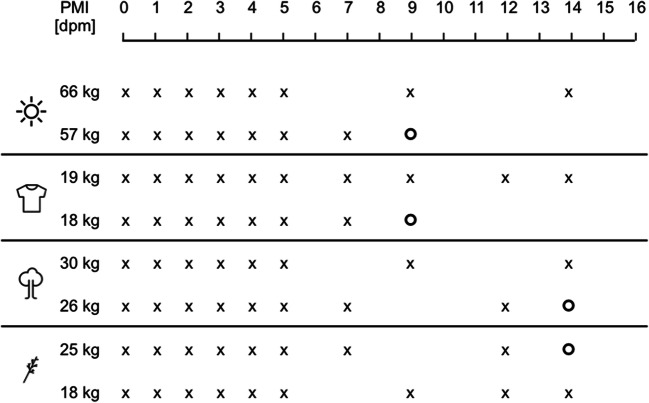
Scheme for obtained tissue samples. “x” marks a sample; “o” marks when no muscle sample was obtainable anymore. No sampling was possible from one clothed (18 kg) and one naked pig (57 kg) from day 9 onwards. On day 14, only one animal from each of the four setups (*n* = 4 in total) provided a sufficient tissue sample. Icons indicate (from top to bottom) open glade naked; open glade clothed; in the shadow of large trees; and covered with branches and twigs

As expected, sampling worked well during the first 5 days postmortem. An appropriate amount of muscle tissue was sampled each time, and the closing of the biopsy opening with super glue was sufficient. With ongoing decomposition, starting after sampling day 7, some of the animals depicted significant morphological changes at the limbs, as the extracted tissue was no longer clearly identifiable as muscle tissue and thus omitted. In some cases, no thigh muscle tissue was present and thus not obtained. Therefore, the sample set for protein analysis had to be adapted accordingly. For days 0 to 5, samples from all animals were included for protein analysis (*n* = 8), while for the following days some samples had to be omitted. The loss of tissue did not correlate with body weight or exposure: after 7 days, the 57 kg pig located in the open glade and the 18 kg clothed pig had to be withdrawn from sampling because of lacking tissue. This applied for the 26-kg pig positioned underneath large trees and the 25-kg pig covered with branches after 12 days. Interestingly, one animal of each group remained to be sampled until day 14. For technical reasons and statistical homogeneity, the sample size was reduced to *n* = 4 for the days 7 to 14 (Fig. 5).

Despite adaptations to the protocols that were used in earlier studies [10], generally, the analyzed proteins depicted typical decomposition patterns, such as the loss of native bands and the occurrence of degradation products at certain time points during the investigated period. Some of the bands appeared blurrier and with some smear compared with previous studies; however, this had no negative effect on data analysis and interpretation. An interesting deviance was that there were no native bands of desmin detectable, using the presented protocol in any of the samples. Desmin degradation products < 35 kDa, however, occurred in all animals at some point.

Tropomyosin appeared as characteristic double bands at day 0 in all animals. Especially in later stages, most of the bands depicted smear between the two bands, making it hard to distinguish between them. Although it appears that one of the two bands disappeared in some of the day 12 and day 14 samples, we refrain from an according interpretation due to a lack of clarity. No tropomyosin degradation products were present in any of the samples.

All day 0 samples depicted clear vinculin and meta-vinculin bands. Meta-vinculin was lost in samples of 6 animals at day 2 and in all 8 animals after day 3. This represented a significant correlation with the PMI (Spearman *ρ* = − 0.641, *p* < 0.001) with a statistic loss of the band at 3.1 days postmortem (dpm). The native vinculin band was lost in 4 animals after day 4, in 6 animals after day 9, and it remained present in two animals until day 14, again representing a significant correlation with the PMI (Spearman *ρ* = − 0.701, *p* < 0.001). However, a large confidence interval for the occurrence of the change arises for the native vinculin band: 0.2–12.9 dpm. Starting on day 1, vinculin degradation products between 100 and 84 kDa appeared. By day 3, all animals depicted a degradation product at 84 kDa. From day 5 and day 9, respectively, a 75 kDa and a 63 kDa degradation product was present in all cases (Spearman *ρ* = 0.661, *p* < 0.001, CI = 0.7–14.8).

Cardiac troponin T was present as a native band of approximately 43 kDa in all day 0 to day 4 samples. At day 5 half, and after day 6, all of the analyzed samples had lost this band. Additionally, a degradation product (38 kDa) was detectable in some samples between day 1 and day 5. After that, no cTnT bands were detectable (Spearman *ρ* = − 0.805, *p* < 0.001, CI = 4.4–5.9).

**Fig. 6:**
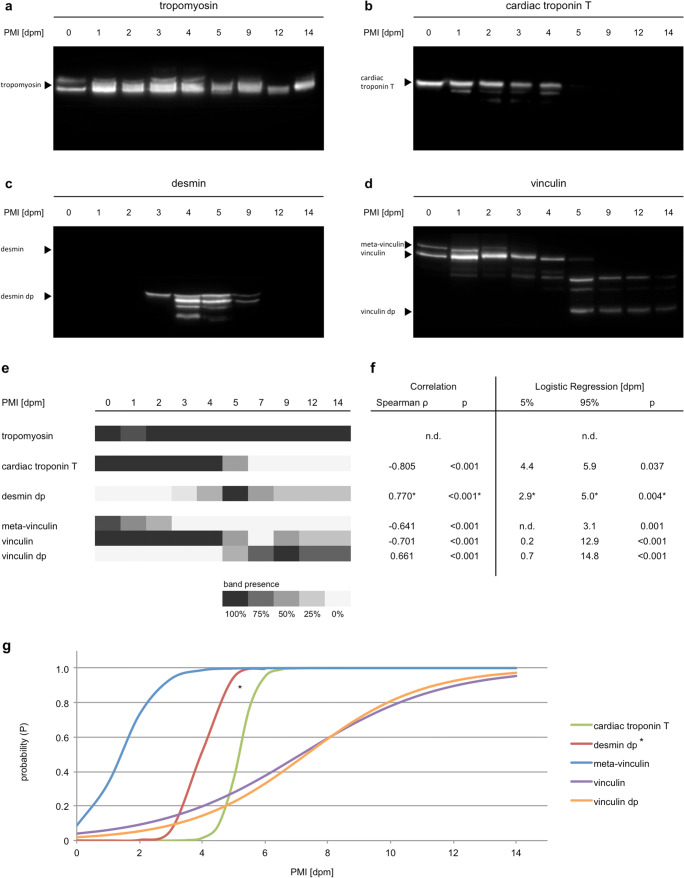
Results of the analysis of muscle protein degradation. **a**–**d** Representative Western blots of skeletal muscle samples throughout the investigated time period of 14 days. **e** Heatmap, depicting the abundance of protein bands (native bands and degradation products (dp)) within all samples. Cardiac troponin T, meta-vinculin, and vinculin depicted a decrease, and vinculin dp an increase of band presence over time. Desmin dp depicted a transient increase until day 5 (100% presence) and a subsequent decrease of abundance. **f**–**g** Statistic analysis of the correlation of protein decomposition with PMI. While some of the changes (loss of native meta-vinculin and cardiac troponin T, as well as the appearance of desmin dp) occur in narrow time frames and short PMIs, others (loss of native vinculin and occurrence of vinculin dp) stretch over an extended time period barely, or not even reach significance levels (95%) within the investigated time period. *Desmin dp statistics were calculated until day 5. The transient character of this degradation product has to be specifically considered

As mentioned earlier, there were no native desmin bands discovered in any of the tested samples. However, between day 3 and day 5, several degradation products (between 38 and 30 kDa) appeared in all animals tested. These degradation products largely disappeared again between day 7 and day 14. The statistical methods applied to the other proteins are not sufficient to be used for this transient characteristic. However, when only the occurrence of degradation products is analyzed until day 5, a significant correlation with the PMI (Spearman *ρ* = 0.770, *p* < 0.001, CI = 2.9–5.0) exists (Fig. 6).

## Discussion

PMI estimation is an important aspect of forensic routine and yet, especially in intermediate and advanced stages of decomposition, often extremely difficult. Postmortem changes or phenomena that are investigated today can be significantly altered by case-specific properties such as individual and environmental conditions. Therefore, knowledge on corrective factors and/or exclusion criteria for each method is crucial for proper application. The availability of multiple approaches and a case-specific selection of the most adequate method (or most adequate combination of methods) potentially provide a modus to increase reliability and/or precision of PMI estimations. In the present pilot study, we were able to characterize strengths and limitations of specific methods under variable conditions and observed substitution, as well as synergetic effects for the use in future case work.

Although outdoor studies per definition underlie (mostly) unpredictable weather conditions, the selected time period and location represented typical Central-European climate from the last decade very well (reference data from the German weather service www.dwd.de, Oct. 2019). Additionally, the changing weather conditions provided some insight in the applicability of certain methods. This makes field studies unique (and thus almost impossible to replicate). However, some factors, such as alternating exposure to solar radiation or changing microclimate and thanato-biodiversity, to which applicable methods for PMI estimation necessarily have to be robust against, can probably never be simulated in a lab. On the other hand, by using animals as a proxy for human decomposition, some other variations, for example, on behalf of individual influencing factors (age, sex, disease, etc.) and especially PMI, can be minimized [38]. Of course, this implicates requiring additional validation studies on humans, but provides exclusive information on the basic principles of decomposition processes. Today, pigs are largely accepted as a reasonable model in forensic sciences and are frequently used for decomposition studies due to their similar physical and biochemical preconditions to humans [37, 39]. Although some variations to human decomposition have been reported [11, 40], pigs generally depict similar morphological changes [41]. This is also supported by the results of this study. Using the scoring criteria of Megyesi et al. that was developed on and for humans [11], the process of decomposition could be well described. More recent studies, specifically addressing changes in pigs, have also shown these qualitative similarities, however detected significant deviations in the temporal occurrence [41]. Difficulties to assess the TBS occurred after day 3, when substantial maggot masses partly impeded assessments. Additionally, to prevent interferences especially with the thanato-biodiversity, we desisted to move (especially to turn over) the animals and to observe the underside of the body. This compromise is, though, common in studies like the present one and should not significantly influence scoring [42].

We did not come across issues with subjective observer variations during this experimental series. Although this can be considered a crucial aspect for reliable scoring (and thus PMI estimation), some basic training of the assessors was sufficient to obtain practically identical data. This was in accordance with a study on subjectivity of TBS scoring methods, reporting reasonable inter-rater reliability [43].

Especially after day 5, the TBS scores of the 8 carcasses began to deviate. On day 12, a maximum difference of 13 points was reached (minimum 18, maximum 32). Using the improved equation for TBS and accumulated degree days provided by Moffat et al. [44], days postmortem (dpm) were calculated. This resulted in estimations of 17.9 dpm (95% CI limits 8.2–40.2 dpm) and 305.1 dpm (95% CI limits 124.9–933.5 dpm), respectively. Despite the possibility of species differences [41], this clearly illustrates the difficulties in the application of this method. As no real trend towards different morphological changes was detected due to factors like body weight and exposure, a multitude of influencing factors and/or extremely large (and thus mostly impractical) confidence intervals have to be concluded in order to use this method for PMI estimation. This becomes even more apparent considering limited individual influences in pigs. Individual differences in humans most certainly additionally complicate application.

During the first 2 days of the experiment, a lot of precipitation and low temperatures around 15 °C impacted and decreased insect activity, hence increasing the probability of a delayed colonization. The difference between the species composition of adult versus larval stages was obvious. To a certain extent, this is not surprising, as numerous succession studies have shown a higher number of species among the adult specimens sampled on or near the carcasses, compared with the larval stages actually developing on the cadaver [45–47]. This can partly be explained by sampling artifacts, within the respective studies (not enough time, etc.), but also because not all adult necrophagous insects found on a cadaver will colonize it, for several reasons, e.g., to avoid competition or because of waiting for a more appropriate later stage of decomposition [48]. Moreover, not all adult insects sampled in the present study show a necrophagous habit in their immature stages, like, e.g., the blow fly *L. bufonivora* [49], a parasite of amphibians or the house fly species of the genus *Morellia*, which are all coprophagous [50]. Hence, they can be rather considered as a random element of carrion insect communities.

Comparing the occurrence of *N. littoralis* (after 2 days) to existing succession data to estimate the time of appearance and period of presence in more detail puts findings into perspective. Matuszewski and colleagues [46, 51, 52] studied 36 pig carcasses at different seasons over 2 years in Polish forests. Two of the four most common species of the present study (viz. *L. caesar* and *N. littoralis*) were also present there, dominated the investigation, and were an important part of the established daily matrix of residency. Using the present findings of the exemplarily chosen “day of discovery” 11 (with the presence of *L. caesar* and *N. littoralis* as adults and larvae) results in an estimated PMI_min_ of 6 (*L. caesar*) and 11 days (*N. littoralis*) and a PMI not longer than 17 (*L. caesar*) and 20 days (*N. littoralis*), respectively [51]. By using this occurrence/presence scheme, the results of the developmental data of *L. caesar* (see above) are thus supported and even maximum possible PMIs are given.

Nevertheless, the unique position of a single species (here *L. caesar*) in a field situation at a PMI of up to 15 days is unusual. The reasons for this can be complex. The majority of entomological samplings worked well, especially for the exposed, naked pigs, but it was much more difficult for the dressed carcasses and the pigs under brushwood. Another limiting factor could be some lack of experience of the collectors. Although the practical training in sample collection, received by the assessors, was adequate for crime scene application, additional preparation towards possible scientific challenges could have been beneficial. This could have led to biased sampling by, e.g., focusing on one type (e.g., the largest and/or most abundant larvae) of insect evidence at the cost of species diversity, or by missing certain life stages, which are more difficult to sample because of their small size or cryptic behavior on the carcass. A resulting low number of species subsequently leads to reduced possibilities for PMI_min_ calculations. For estimations and reports, caution is required when applying succession and so-called pre-appearance data, the latter mainly introduced by Matuszewski and colleagues in the last decade [37, 46, 51, 52]. Such data are in the same way (or even more) impacted by a professional, standardized sampling of insects. One could also argue or speculate that some conclusions about the arrival and presence time of certain species are not sufficiently based on hard data or experiments. Whatever reason, the findings based on published data on the pre-appearance interval of the carrion beetle *N. littoralis* are confusing, as they indicate an exaggerated PMI_min_. Whether this is due to a weak performance of the underlying model or other reasons (e.g., population-specific differences in development or climatic preferences) remains unclear. However, both most important taxa in this field study, the blow fly *L. caesar* and the carrion beetle *N. littoralis*, share one thing in common—the lack of reference data for development. There are still blank spots on the map, i.e., insect species of forensic relevance but without any developmental data.

Altogether this study shows some problematic issues in forensic entomology: (i) possibly vague data bases or no data at all for the sampled taxa, (ii) the dependency on good insect sampling on site, and (iii) the diagnostic gap, i.e., the fact that there can be a delayed colonization, causes the entomological clock to start running one to several days later.

Qualitative changes of protein band patterns such as occurring degradation products or the loss of native protein bands can be valuable clues for PMI estimation. However, to date, there is only limited reference data for humans [26] and available data regarding the porcine animal model exclusively originate from controlled laboratory studies [10]. To provide further reference data, we tested the method in uncontrolled field conditions by comparing results of this animal study to already available reference data from controlled environments. To additionally improve the applicability of the method in field conditions, we were able to adapt sampling and sample processing for routine practice.

Comparing degradation patterns of tropomyosin, this protein turned out to be very stable towards decomposition, as it has been shown previously for humans and pigs [10, 26]. The significant loss of native cardiac troponin T (cTnT) after day 5, in comparison, partly contradicts previously described results in pigs, where this change occurred after 9 to 10 days postmortem at 21 °C under controlled laboratory conditions [10]. The exact reason why no native bands of desmin were detectable remains unclear. This deviation from usually seen results can be due to adaptations of sample processing to enable efficient application of the method in field conditions. Instead of fixing the biopsy samples with liquid nitrogen, which should be the method of choice to immediately end autolytic processes and thus preserve the degradation status, a chemical preservation with RIPA buffer including protease inhibitor was used. Although this protocol obviously had no effect on the detection of other proteins, a negative reaction with native desmin cannot be excluded and remains to be further tested. As far as valid interpretation is possible, the absence of native desmin did not affect the occurrence of desmin degradation products. While desmin fragments were detected in all samples with increasing frequency from day 3 to day 5, similar degradation products have been reported after 1 to 2 days in pigs stored at 21 °C [10] and after a few hours in humans when ADD is back-calculated to PMI with 20 °C assumed [26]. Divergences such as temporal deviations of absence or presence of certain bands can be partly explained by a lower mean temperature in the field experiment (just over 17 °C during the first 5 days). The much higher humidity (usually near saturation due to regular rainfalls vs. 35 ± 5% relative humidity in lab experiments) in combination with the fact that in lab experiments explanted legs were used (drying is promoted by large areas without protecting skin) might also cause altered temporal degradation pattern as seen for cTnT. A further interference factor was represented by increased insect activity with a consequent excessive loss of tissue and thus unavailability to analyze proteins. Notably, all samples taken after day 5 (*n* = 4) depicted low protein concentrations and comparably weak bands in all proteins analyzed. In the case of cTnT, together with a high humidity, this could explain the earlier loss of native bands in comparison with previous lab experiments.

In practice, the disappearance of native proteins and protein fragments depicts only a minor problem for statistical modeling or the application for PMI estimation, as it could only be applied for present degradation products. If no degradation products (e.g., of desmin) were detected in a certain case scenario, other proteins would have to be used to conclude a PMI (e.g., cTnT). The phenomenon of transiently occurring degradation products as it was found for desmin fragments in this study, however, has to be considered for fragments of other proteins as well, thus underling the importance of positive control proteins (such as tropomyosin).

To our knowledge, this is the first experimental series analyzing vinculin degradation in pigs on a quantitative basis. Indeed, the present results show the potential of this protein for future application in PMI estimations. Especially, the loss of the meta-vinculin band, occurring at day 3 in all animals, confirms the results of previous pilot experiments on pigs and humans [53] and quantitative experiments on rats [54]. Also, the loss of native vinculin and the appearance of degradation products have been reported [53, 54]. However, in this particular study, these changes occurred in the later stages of the experimental series in which only a small sample size was available, thus producing large confidence intervals. Additional experiments are necessary to test the applicability for future practice. The results of this experiment depict the potential of muscle tissue (protein) degradation analysis for PMI estimation, particularly within the first 5 to 10 days. This is especially underlined by the finding that protein degradation was largely robust towards the individual influencing factors body weight and exposure. However, at the same time, differences to previous results show the importance to further investigate the effect of factors such as temperature and humidity in detail.

## Conclusion

This pilot field study partly met the objectives to compare the applicability of methods for PMI estimation in advanced decomposition stages. Strengths, weaknesses, and limitations of morphological scoring methods (TBS), tissue decomposition (skeletal muscle protein degradation), and forensic entomology were clearly revealed, underlining the current restrictions and future challenges in this field. Morphological methods and skeletal muscle protein degradation produced fairly consistent data within the first 5 days. Afterwards, morphological changes were found to be highly variable upon individual influences, even under the same environmental conditions. Protein analysis was largely robust to individual influences; however, it could no longer be applied in some of the carcasses after day 7 due to indirect environmental influences (promoting insect colonization and a resulting loss of tissue). When tissue was preserved, due to less insect activity, protein analysis provided valuable results even until day 14. On the contrary, from carcasses lacking tissue after a couple of days, a sufficient amount of fly specimens was available for forensic entomological analysis and the estimation of a minimum PMI (PMI_min_), demonstrating a valuable complementary effect of entomology and protein analysis. In this field study, forensic entomology could not provide evident data in the early postmortem period, but became increasingly important, and ultimately the last sufficiently applicable approach. Valuable PMI_min_ estimations were obtained mainly by analysis of larval stages of blow flies. However, to date, no reliable reference data exists for some of the most abundant species in this study. In practical case work, a recommended approach might be to use data from closely related species. However, it has to be ensured that both the species (one from which the reference data derived and one for which calculations are be made) have similar geographic origins [55].

A combination of the applied methods could not increase the overall accuracy of PMI estimations, but having various techniques available covered a significantly larger time period compared with an individual approach. Future studies on all three methods are required to (i) investigate (additional) influencing factors, (ii) increase reference data, and (iii) further investigate respective interactions in both, the animal model and humans, to be able to provide a sufficient tool-box for a broad application spectrum in forensic case work.
